# Cyclic Behavior of Masonry Shear Walls Retrofitted with Engineered Cementitious Composite and Pseudoelastic Shape Memory Alloy

**DOI:** 10.3390/s22020511

**Published:** 2022-01-10

**Authors:** Alireza Tabrizikahou, Mieczysław Kuczma, Magdalena Łasecka-Plura, Ehsan Noroozinejad Farsangi

**Affiliations:** 1 Institute of Building Engineering, Poznan University of Technology, Piotrowo 5, 60-965 Poznan, Poland; mieczyslaw.kuczma@put.poznan.pl; 2 Institute of Structural Analysis, Poznan University of Technology, Piotrowo 5, 60-965 Poznan, Poland; magdalena.lasecka-plura@put.poznan.pl; 3 Faculty of Civil and Surveying Engineering, Graduate University of Advanced Technology, Kerman 7631818356, Iran

**Keywords:** masonry wall, retrofitting, engineered cementitious composite, shape memory alloy, numerical analysis, Abaqus

## Abstract

The behavior of masonry shear walls reinforced with pseudoelastic Ni–Ti shape memory alloy (SMA) strips and engineered cementitious composite (ECC) sheets is the main focus of this paper. The walls were subjected to quasi-static cyclic in-plane loads and evaluated by using Abaqus. Eight cases of strengthening of masonry walls were investigated. Three masonry walls were strengthened with different thicknesses of ECC sheets using epoxy as adhesion, three walls were reinforced with different thicknesses of Ni–Ti strips in a cross form bonded to both the surfaces of the wall, and one was utilized as a reference wall without any reinforcing element. The final concept was a hybrid of strengthening methods in which the Ni–Ti strips were embedded in ECC sheets. The effect of mesh density on analytical outcomes is also discussed. A parameterized analysis was conducted to examine the influence of various variables such as the thickness of the Ni–Ti strips and that of ECC sheets. The results show that using the ECC sheet in combination with pseudoelastic Ni–Ti SMA strips enhances the energy absorption capacity and stiffness of masonry walls, demonstrating its efficacy as a reinforcing method.

## 1. Introduction

Seismic activities can induce structural and non-structural damage both during and afterwards the event, which is mainly caused by perturbations in load-resisting elements of constructions, such as load-bearing walls and columns [1,2]. In most cases, seismic failures in structures are triggered by their limited resilience owing to deficient component size, material properties, and lack of structural flexibility or ductility [3,4]. When subjected to earthquake loadings, brittle construction materials such as unreinforced concrete and unreinforced masonry (URM) do not display sufficient ductility [5,6]. As a result, engineers have identified retrofitting URM-based structures as a critical challenge that should be resolved.

The mass of the wall plays a key role in determining the out-of-plane performance and strength of the wall by minimizing the bending moment transmitted to the wall out-of-plane. However, the in-plane applied loads dictate the cyclic behavior and seismic strength of the URM walls [7]. As a result, the authors’ main objective in this work was to evaluate the in-plane behavior of URM walls under cyclic loadings.

The first structural seismic retrofitting method for URM structures was the application of timber components in the construction of URM walls in ancient Greece following seismic occurrences [8]. Porto et al. [9] investigated the in-plane behavior of masonry walls using a pair of parallel and vertical direction punctured components based on their analysis on fracture growth, energy absorption capacity, viscous damping, and other parameters. Gouveia and Lourenco [10] evaluated 16 distinct URM walls that were equipped with coils and subjected to cyclic loads. The technique they employed improved the diagonal resistance and stiffness of the URM walls by up to 30%.

Conventional strengthening processes can fail to provide construction with substantial strength to the maximal expected seismicity, and may result in culturally undesirable alterations to the initial structural design [11]. Reinforcement employing these methods is usually complex, resulting in interruption of utilization, higher budgetary demands, and, in some cases, struggling to maintain them [12].

Other innovative retrofitting methods, such as using fiber-reinforced plastic (FRP) materials and metal jackets, might have the following disadvantages:

- Steel jacketing: the tendency to corrode and the complexity of installation by machines. The infilled region, which is the space among both the cementitious gap and the steel jacket, becomes clogged, causing pillar irregularity [13].
- FRP materials can have a wide range of Young’s modulus depending on their composition. Carbon-FRP (CFRP) materials, for example, can have elastic modulus ranging from 37 to 784 GPa, whereas Glass-FRP (GFRP) materials can have elastic modulus varying from 35 to 86 GPa [14]. They may, however, have an overall lack of ductility and shear strength [15].

To retrofit the masonry constructions, other technologies and materials, such as SMA, can be implemented [16,17,18,19,20]. SMAs exhibit many unusual properties, in particular the ability to recover to their former configuration after being exposed to severe deformations. This is a result of a martensitic phase transformation generated through either heating (defined as the shape memory effect) or removing the applied load (defined as superelasticity or pseudoelasticity) [21,22,23]. Among many materials that exhibit shape memory behavior, the Ni–Ti alloy (known as nitinol) thus far has demonstrated an outstanding behavior, with large shape recovery, recovery stress, and superelastic strain [24,25]. Compared to stainless steel, which is one of the most extensively used materials in building engineering, Ni–Ti has outstanding features, as shown in Table 1.

Casciati and Hamdaoui [27] investigated the use of connected devices based on the pre-tensioned SMA wires in the rehabilitation of historic masonry buildings. The results of the experimental study were used to develop a numerical model that takes into account the impacts of SMA devices. The structure was first analyzed in its pristine configuration, with no retrofitting procedures, and then the impacts of various retrofitting procedures were validated experimentally and included in the numerical analysis by adjusting the equivalent Young’s modulus correspondingly.

Cardone et al. [28] proposed a technique for improving the seismic behavior of steel-based joints in historic structures through the application of pre-tensioned copper-based SMA cables. Testing outcomes show that the suggested mechanism is effective at reducing force differences caused by changes in air temperature. The test results reveal that the proposed SMA-based device is extremely successful in improving the thermal behavior of steel connections. Furthermore, the force fluctuations in steel tie-rods caused by changes in air temperature are 80–90% lower with SMAs than without.

Rezapour et al. [29] investigated the performance of URM walls strengthened with iron-based SMA strips installed in the configuration of crossovers and parallels in brickwork and exposed to post-tension stress. According to the findings of this study, the stiffness increased by 98.1% in the vertical-strip walls and by 127.9% in the crossover model’s position. Furthermore, the greatest resistance in the parallels arrangement was 108 kN, but by the end cycle, it had been reduced by more than half to 40 kN.

Habieb et al. [30] investigated the usage of integrated SMA-based cables with a tensioned fiber-reinforced rubber isolator as the base isolation system for earthquake resistance of an ancient cathedral. Because of its significant energy absorption capabilities, the recommended model with a 2% pre-strain SMA wire model exhibits the greatest decrease of the church’s transverse deformations and greatly reduces impact (from destruction to light damage level) in the case of major earthquakes.

Another unique method for retrofitting URM structures is the use of ECC technology, which first appeared in the early 1990s. The ECC is a fiber-cement-based composite material with significant cyclical ductility.

Kyriakides et al. [31] used a finite element macro-modeling technique to investigate the performance of a URM beam retrofitted with a thin coating of ECC. The void of the masonry junction, fracturing of the ECC substrate under the mortar joints, and disintegration of the ECC were all fully demonstrated. A three-fold-quicker erosion in the ECC model’s response resulted in a 40% loss in ductility of the refurbished girder, and an 8% reduction in ECC flexural capability, resulting in a 17% drop in retrofitted masonry beam strength.

Gencturk and Hosseini [32] conducted extensive studies to measure the energy absorption capability of concrete buildings reinforced with ECC under seismic loadings. The stiffness, strength, ductility, and energy absorption capacity of columns composed of various ECC combinations were found to be 110, 65, 45, and 100% greater, respectively, than those of reinforced concrete columns under cyclic loading.

Munjal and Singh [33] investigated the out-of-plane flexural behavior of URM walls reinforced with ECC sheets. The load-carrying capability of torsional reinforced masonry walls with prefabricated ECC sheet was found to be 440% more than that of control/unstrengthened masonry walls.

Singh et al. [34] investigated the out-of-plane performance of a URM sandwich-like ECC-based beam coupled with epoxy material. It was discovered that using prefabricated ECC boosts the stiffness and deformability of masonry beams.

Singh and Munjal [35] used numerical simulation to conduct a parametric analysis of the out-of-plane performance of ECC-based URM walls with openings. They discovered that the load-carrying capability of ECC-enhanced masonry walls with apertures is approximately six times that of the examined unstrengthened masonry walls.

To evaluate the implementation of Ni–Ti SMAs and ECC sheets to enhance the performance of masonry walls, finite element models of a number of masonry walls reinforced with Ni–Ti strips and ECC sheets of various thicknesses, as well as a combination of both methods, are described in the current study. All models were subjected to quasi-static cyclic loading, and their behavior was assessed. The research shows that it is beneficial to retrofit masonry structures with Ni–Ti strips and precast ECC sheets, as well as combining both technologies to improve the load-bearing capacity to withstand cyclic loads.

## 2. Computational Models

**Figure 1:**
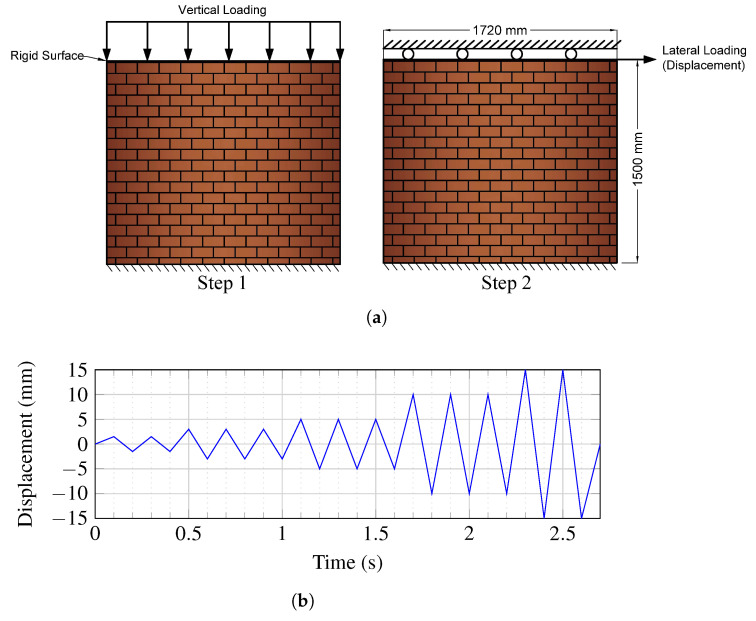
(**a**) Schematic of applying the vertical and horizontal loading and displacement; (**b**) the amplitude of applied lateral displacement.

The geometrics of the numerically modeled brick wall were based on the study by Karimi et al. [36] and had a height of 1.5 m, a length of 1.72 m, and a thickness of 19.5 cm (Figure 1a). To replicate gravity loads applied to brick walls, constant vertical pressure of 0.2 MPa is imposed as the control unit on the top of the specimens through a rigid surface. To model the earthquake-induced behavior, lateral in-plane cycle loads are employed as displacement control on the top of the wall [37]. The amplitude of lateral cyclic displacements is increased gradually on the sample, and each phase is repeated three times (Figure 1b). The authors used the material properties of the bricks in the Concrete Damage Plasticity (CDP) model experimentally tested and provided by Munjal and Singh [33]. Commercial finite element software Abaqus was used in this study to examine the effect of retrofitting with SMA strips or/and ECC sheets on the cyclic behavior of URMs.

**Figure 2:**
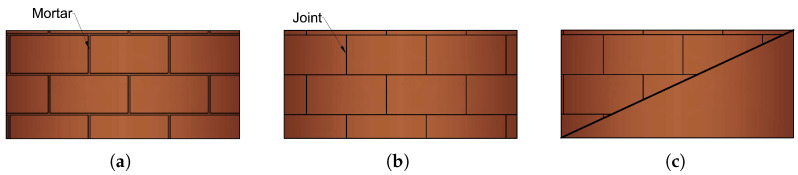
Different approaches to numerical modeling of masonry walls; (**a**) micro-model; (**b**) simplified micro-model; (**c**) macro-model.

Masonry prism can be modeled in three different methods, as follows:

- Detailed-microscopic: The bricks, mortar and the interaction between them is fully modeled (Figure 2a).
- Simplified-microscopic: The mortar is modeled as cohesive interaction between bricks (Figure 2b).
- Macroscopic: The whole brickwork prism is modeled as a combination of interconnected, homogenous, and fragile solid (Figure 2c).

The micro-modeling approach is the most accurate, especially for small structural elements where the interface involving bricks and mortar is of critical priority [38]. This is also significant when the emphasis is on the specifics of local distributions of stresses and damage, while, when only the general functionality of brickwork is examined, specifically the push-over analysis, the macro-modeling approach produces accurate results [39]. Bricks and junctions are linked in a homogeneous format in a macro-modeling approach, and a relationship between mean masonry stresses and strains is provided. The computing complexity is another essential parameter that must be addressed when comparing modeling methodologies [40]. Consequently, the micro-modeling technique is by far the most demanding, but the macro-modeling approach provides a reasonable balance of precision, numerical efficiency, and computation complexity, especially when simultaneous computing is applied. As a result, the most appropriate technique is highly associated with the exact topology of masonry structures to be evaluated as well as the analysis’s purposes [41].

Although the crack-growing processes in the URM cannot be locally and precisely determined by using the macroscopic modeling approach, the principal benefit of it is that the computational algorithms are significantly faster. Furthermore, macroscopic modeling has shown proper results in terms of overall behaviors, such as base shear force [42]. Therefore, in this work, as an approximation of the complex nonlinear problem under study, macroscopic modeling was used to simulate the cyclic response of the retrofitted URMs in the finite element software Abaqus.

**Figure 3:**
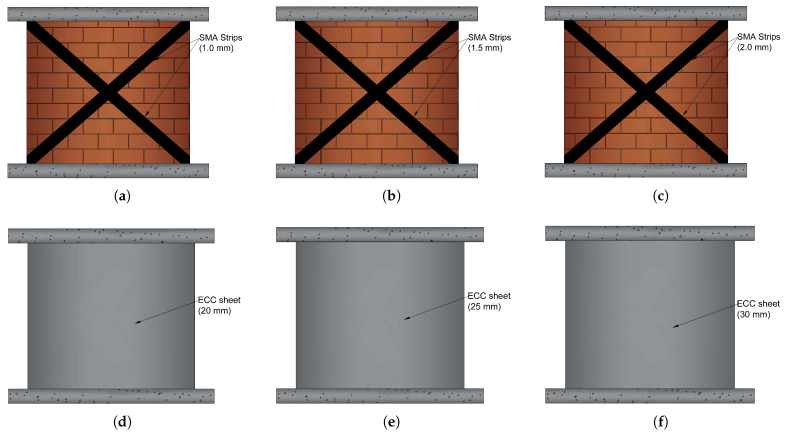
Numerically studied walls; (**a**) URM-SMA-1.0 mm; (**b**) URM-SMA-1.5 mm; (**c**) URM-SMA-2.0 mm; (**d**) URM-ECC-20 mm; (**e**) URM-ECC-25 mm; (**f**) URM-ECC-30 mm.

**Figure 4:**
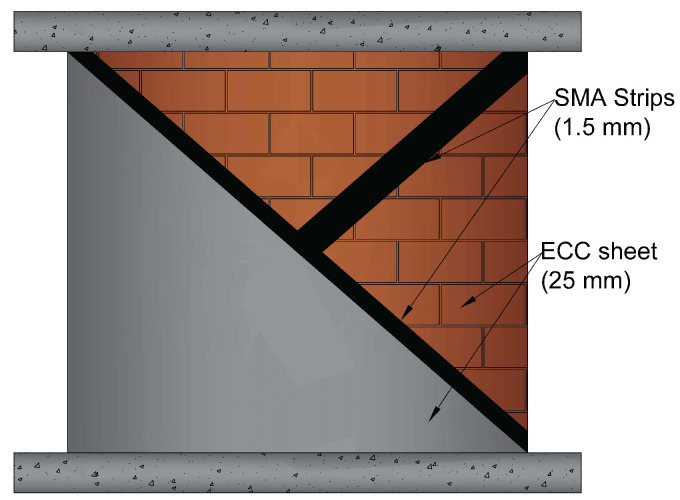
The numerically studied hybrid URM strengthening method using Ni–Ti strips and ECC sheets.

In this study, several retrofitting techniques based on Ni–Ti strips and ECC sheets were used. In the first technique, Ni–Ti strips are attached on both sides of a URM in the shape of an X-bracing with three distinct thickness values. The ECC sheets with three different thicknesses are bonded on both sides of the URM in the second technique. The last technique examines the combination of these techniques. Figure 3 and Figure 4 illustrate the schematic of these models and Table 2 provides the geometrical information on them. As the dimensions of the wall in all models are as shown previously in Figure 1a with the same height, length, and thickness as 1720mm×1500mm×195mm, respectively, only the geometrics of the additional reinforcing elements are presented.

The Ni–Ti strips are entirely embedded in the ECC sheets in the hybrid reinforcing model, and the sheets then cover the whole two sides of the wall. However, in Figure 4, to highlight the model’s reinforcing components, the ECC sheet is depicted as covering half of the wall, although in the actual model it has the same height and length as the wall.

### 2.1. Material Model

The mechanical parameters provided by Pereiro-Barceló and Bonet [43] for the Ni–Ti material utilized as strips in the numerical modeling were used in this study to investigate the influence of SMA-based retrofitting on URM walls. Table 3 displays these mechanical properties.

**Figure 5:**
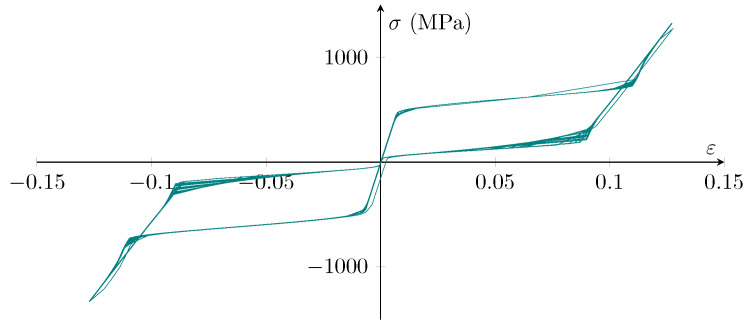
The stress–strain curves of the Abaqus SMA material model proposed by Auricchio [44,45,46] and the properties provided by Pereiro-Barceló and Bonet [43].

To control the materials implementation accuracy in Abaqus, the provided data in Table 3 were used to model a 40 mm × 40 mm SMA shell element. The mesh size for this element was 2 mm with a quad element shape with the medial axis algorithm. Figure 5 depicts the stress–strain curves derived using Abaqus. When the findings are compared to the hysteresis curves reported by Pereiro-Barceló and Bonet, it is apparent that the material model functions efficiently in the current Abaqus program.

The mechanical behavior of masonry walls is considered to be isotropic and homogeneous, equivalent to that of concrete, but with different material strengths while using the macro-modeling method. As a result, the CDP model developed by Lubliner [47], Lee and Fenves [48], was employed for numerical simulations of the quasi-brittle materials.

The modulus of elasticity of masonry and ECC used for analytical modeling are 1450 and 17,500 MPa, respectively, based on experimental results provided by Munjal and Singh [33]. The material parameters required for the CDP model were specified to characterize the plasticity values of the masonry prism in Abaqus, as indicated in Table 4.

$\sigma_{b0}/\sigma_{c0}$ is a ratio of the strength in the biaxial state to the strength in the uniaxial state. The dilation angle (ψ) determines the dilatancy under high confining pressure whose value of 30${}^{\circ }$ is selected based on Agnihotri et al. [49]. The flow potential eccentricity defines the rate at which the hyperbolic flow capacity approaches its equilibrium state (ε). At preliminary yield, $K_{c}$ is the ratio of the tensile meridian’s secondary stress invariant to the compressive meridian’s second stress invariant, and it must meet the criterion $0.5<K_{c}\leq 1$ for any known amount of the stress invariant such that the greatest primary stress is negative. One of the most common convergence failures in computational analytic software is attributed to material softening and stiffness degradation, which may be efficiently processed by establishing viscoplastic parameterization by specifying positive tangent stiffness values in relatively small period intervals (μ).

**Figure 6:**
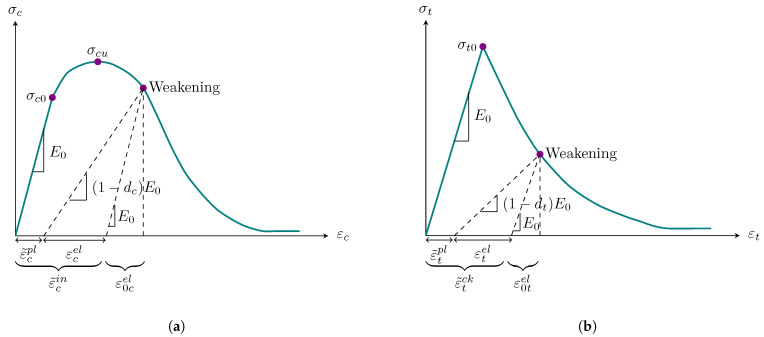
Uniaxial stress–strain relationship: (**a**) compression; (**b**) tension (Abaqus manual [50]).

A uniaxial stress–strain relationship is depicted in Figure 6 to represent the hardening and decline in stiffness of the masonry prism when exposed to compressive and tensile loading.

The compressive hardening (${\tilde{\varepsilon }}_{c}^{in}$) and tensile cracking (${\tilde{\varepsilon }}_{t}^{ck}$) strains are calculated by subtracting from the total cumulative strain the elastic strain that relates to intact specimen:

(1) $${\tilde{\varepsilon }}_{c}^{in}=\varepsilon_{c}-\varepsilon_{0c}^{el}$$

(2) $${\tilde{\varepsilon }}_{t}^{ck}=\varepsilon_{t}-\varepsilon_{0t}^{el}$$

where $\varepsilon_{c}$ and $\varepsilon_{t}$ are total compressive and tensile strains, respectively. In Equations (1) and (2), $\varepsilon_{0c}^{el}$ and $\varepsilon_{0t}^{el}$ are compressive and tensile elastic strains, respectively, which relate to intact material and can be calculated by Equations (3) and (4) (see Figure 6).

(3) $$\varepsilon_{0c}^{el}=\frac{\sigma_{c}}{E_{0}}$$

(4) $$\varepsilon_{0t}^{el}=\frac{\sigma_{t}}{E_{0}}$$

Furthermore, compressive (${\tilde{\varepsilon }}_{c}^{pl}$) and tensile (${\tilde{\varepsilon }}_{t}^{pl}$) plastic strains can be determined calculated by the formulae

(5) $${\tilde{\varepsilon }}_{c}^{pl}={\tilde{\varepsilon }}_{c}^{in}-\frac{d_{c}}{\left(1-d_{c}\right)}\frac{\sigma_{c}}{E_{0}}$$

(6) $${\tilde{\varepsilon }}_{t}^{pl}={\tilde{\varepsilon }}_{t}^{ck}-\frac{d_{t}}{\left(1-d_{t}\right)}\frac{\sigma_{t}}{E_{0}}$$

in which $d_{c}$ and $d_{t}$ are compressive and tensile uniaxial softening coefficients, being measures of damage development, and are computed by Equations (7) and (8).

(7) $$d_{c}=1-\frac{\sigma_{c}}{\sigma_{cu}}$$

(8) $$d_{t}=1-\frac{\sigma_{t}}{\sigma_{t0}}$$

where $\sigma_{t0}$ and $\sigma_{cu}$ are maximal tensile and compressive yield stresses, respectively.

The stress–strain relationships under uniaxial tension and compression loading are as follows:

(9) $$\sigma_{t}=\left(1-d_{t}\right)E_{0}\left(\varepsilon_{t}-{\tilde{\varepsilon }}_{t}^{pl}\right)$$

(10) $$\sigma_{c}=\left(1-d_{c}\right)E_{0}\left(\varepsilon_{c}-{\tilde{\varepsilon }}_{c}^{pl}\right)$$

The material properties (yield stress versus inelastic/cracking strain) used in the numerical modeling were obtained from material characterization tests performed by Munjal and Singh [34] and are listed in Table 5, except for the following assumptions:

- The masonry tensile strength is taken as 10% of its measured compressive strength [51].
- The Poisson’s ratio of the masonry is assumed to be 0.2 [52].

### 2.2. Interactions

**Figure 7:**
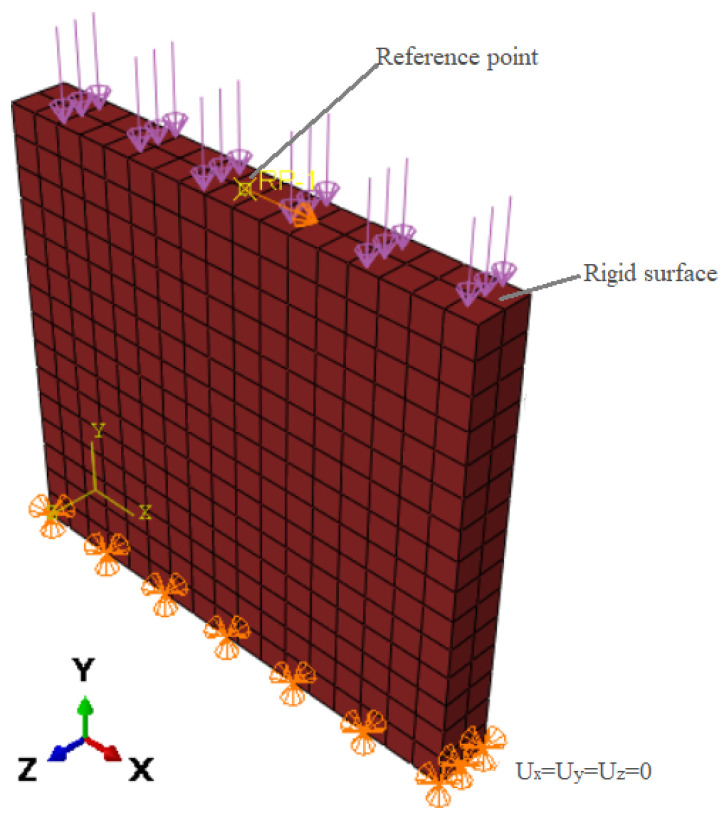
The constraints and interactions applied in the numerical modeling of the investigated URM.

Because the masonry wall was fastened from both edges to a steel girder in the experimental test, and because the stiffness of this beam is significantly higher than that of the masonry prism, the top surface is considered to be rigid to lower the analysis time and DoF of the model. The constraints and interactions for this analysis were defined in Abaqus. The top side of the brick wall was fixed to a datum point in the middle of the top of the wall to establish a rigid surface on top of it—i.e., the displacements of all the finite element mesh nodes on this plane are equal to the movement of the reference point (Figure 7).

**Figure 8:**
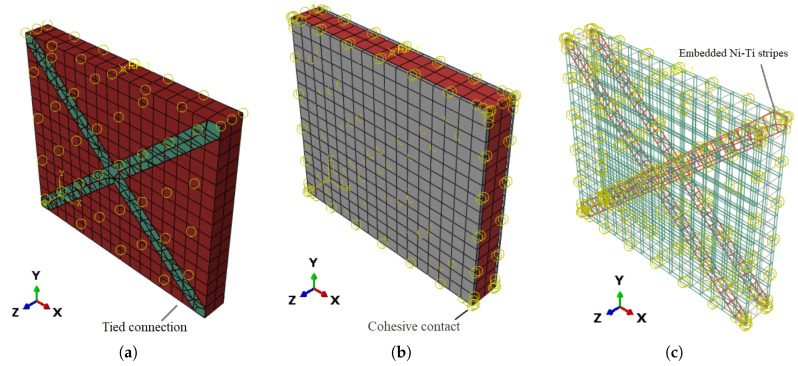
Interactions between different parts and the URM; (**a**) Ni–Ti strips; (**b**) ECC sheets; (**c**) embedded Ni–Ti strips in the ECC sheets.

The interconnection between the masonry wall and the Ni–Ti strips was considered to be mortar-bonded. This approach of interaction binds the strips to the wall while also allowing for the entire transmission of displacements and forces from the strip to the wall (Figure 8a).

The ECC plate was cemented to the brick wall with resin, resulting in surface-to-surface interaction with cohesive behavior (Figure 8b). The constitutive relation shown in Equation (11) was utilized to create the model’s interaction depending on shear and normal stresses.

(11) $$t=\left\{\begin{array}{c} t_{n}\\ t_{s}\\ t_{t} \end{array}\right\}=\left[\begin{array}{ccc} K_{nn} & K_{ns} & K_{nt}\\ K_{ns} & K_{ss} & K_{st}\\ K_{nt} & K_{st} & K_{tt} \end{array}\right]\left\{\begin{array}{c} \delta_{n}\\ \delta_{s}\\ \delta_{t} \end{array}\right\}=K\delta$$

where t denotes the hypothetical friction force, δ indicates the equivalent detachment, and the sub-indexes *n*, *s*, and *t* denote the normal, shear, and tangential components, respectively. The value of the stiffness ($K_{i}$) employed for the numerical simulation is calculated by Equation (12).

(12) $$K_{i}\geq \alpha \times \frac{E_{i}}{t_{a}}$$

where $K_{i}$ and $E_{i}$ are stiffness and Young’s modulus in normal and in-plane directions, $t_{a}$ is cohesive element thickness, and α is a variable whose magnitude must be greater than 1 [53]. The normal ($K_{nn}$), shear ($K_{ss}$), and tangential ($K_{tt}$) stiffness components are regarded to be comparable ($K_{nn}=K_{ss}=K_{tt}$). The stiffness of the cohesive contact is determined by Equation (12) to be 28,000 MPa.

In the hybrid retrofitting method, the Ni–Ti strips were embedded in the ECC sheets as shown in Figure 8c.

### 2.3. Validation of Numerical Model and Mesh Sensitivity Analysis

**Figure 9:**
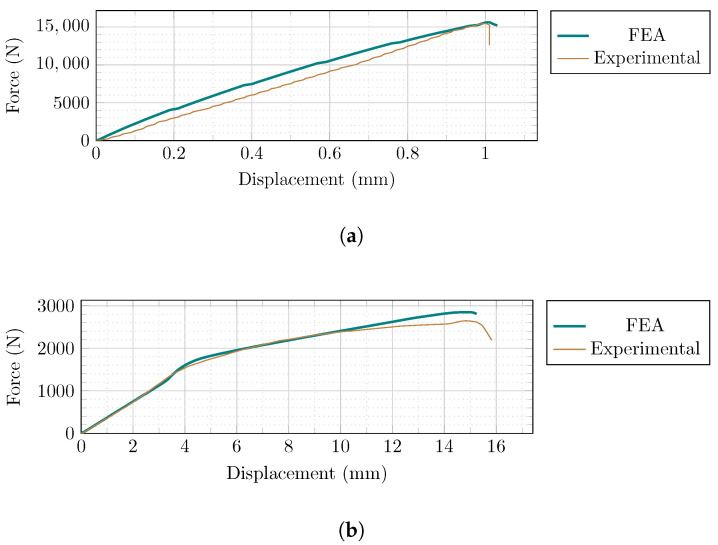
Comparison between the finite element analysis and the experimental results; (**a**) masonry prism; (**b**) ECC sheets.

A finite element analysis (FEA) is conducted to simulate the laboratory experiments that were carried out by Singh and Munjal [35]. Table 6 compares the results we obtained in the numerical modeling and the experimental studies, and Figure 9 depicts both the load-displacement curves. Based on these data, the suggested model provides a satisfactory approximation when compared to the measured values in experimental investigation.

**Figure 10:**
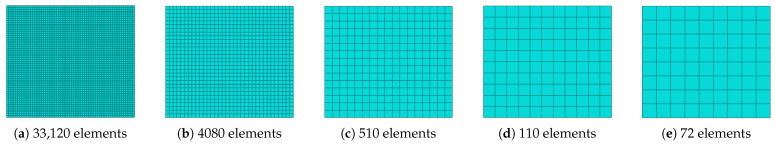
Mesh sensitivity analysis with different mesh sizes and element numbers: (**a**) 25 mm; (**b**) 50 mm; (**c**) 100 mm; (**d**) 150 mm; (**e**) 200 mm.

Mesh sizes of 200, 150, 100, 50, and 25 mm, as well as quadratic element (C3D20) and three-dimensional cubic element with eight points and reduced integration (C3D8R), were utilized to examine the relationship between force and the displacement at the assigned reference point to monitor the model’s mesh dependency (Figure 10).

**Figure 11:**
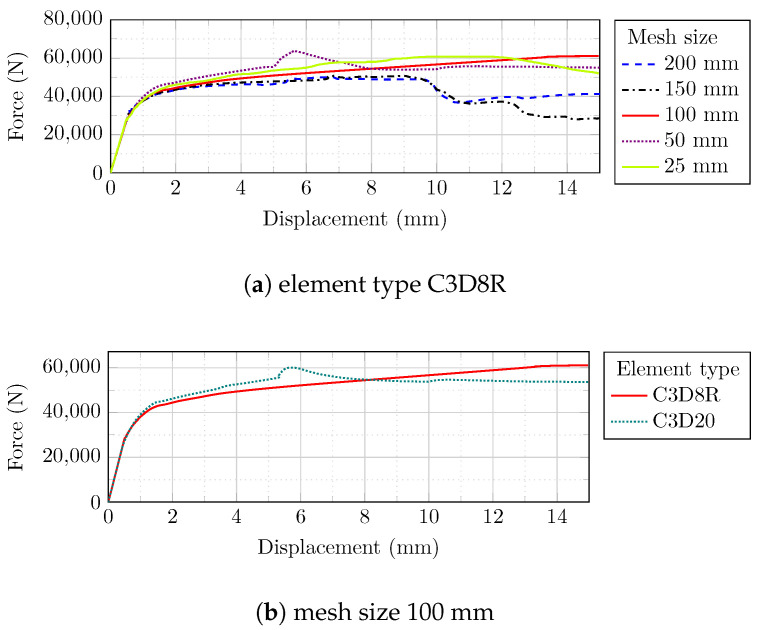
Mesh sensitivity analysis of the masonry prism: (**a**) different mesh sizes; (**b**) different element types.

The cyclic behavior of the masonry prism is readily converged by reducing the mesh size from 200 to 100 mm, while further mesh size reductions (to 50 and 25 mm) did not result in a better stabilization tendency (Figure 11a). Furthermore, altering the element type from C3D8R to C3D20 extended the analysis running time significantly (from 0.5 to 10.8 min, respectively), whereas the C3D8R element type resulted in a satisfactory response and substantially less analysis time (Figure 11b).

**Figure 12:**
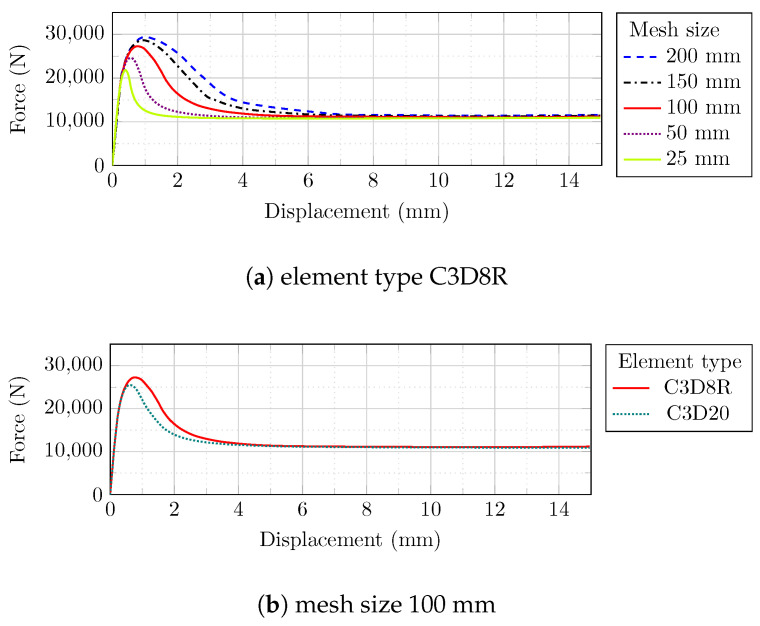
Mesh sensitivity analysis of the ECC sheet: (**a**) different mesh sizes; (**b**) different element types.

A mesh sensitivity analysis of the ECC sheet revealed that a mesh size of 100 mm with C3D8R element generated superior stabilizing characteristics under cyclic loadings (Figure 12).

### 2.4. Meshing and Boundary Conditions

Based on the results of the mesh sensitivity analysis, the C3D8R element with a mesh size of 100 mm was applied for numerical simulations of the masonry prism and ECC sheet, respectively. Additionally, Ni–Ti strips are modeled with a four-node shell element with reduced integration (S4R).

The boundary conditions and constraints applied in the current study are defined based on the experimentally tested masonry wall by Karimi et al. [36], as shown previously in Figure 1.

Two beams placed at the base and the top of the masonry wall are deemed as rigid surfaces. They represent the foundation of the wall and its upper parts, respectively. Therefore, the foundation cannot move in three dimensions in the numerical simulations, and the top surface is considered to be bound as a rigid body pinned to the reference point. The reference point in the center of the upper end of the wall is subjected to lateral cyclic loading and uniform compression of 0.2 MPa on the upper side of the wall.

## 3. Numerical Analysis Results

The behavior of masonry walls strengthened with ECC sheets, Ni–Ti strips, and a hybrid combination of these two methods was examined under cyclic loadings.

First, it is essential to note that the numerical parameters utilized in this analysis were previously validated in the Munjal and Singh research work [33]. The behavior of URM is comparable to the empirical performance, with the highest divergence of 0.21% in displacements and 1.48% in maximum stresses. As a consequence, it is confirmed that numerical modeling of URM for cyclic behavior studies yields adequate outcomes that are approximate to relevant practical data.

**Figure 13:**
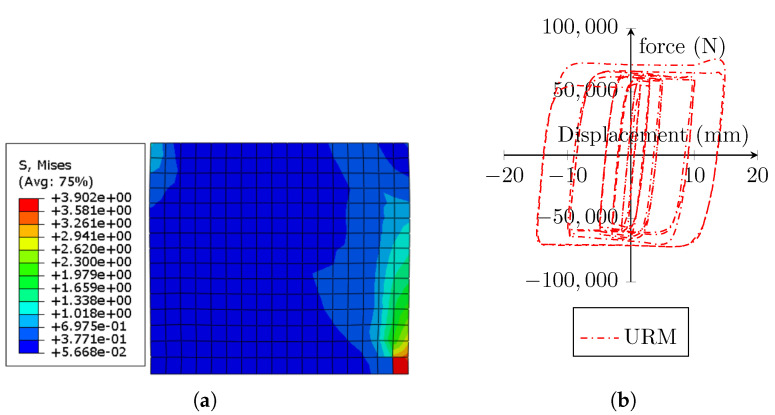
(**a**) Stress distribution in the URM model; (**b**) hysteresis of the URM.

Furthermore, an unreinforced masonry wall with the previously specified details was studied. Figure 13a depicts the stress concentration in the URM at the completion of the imposed load cycles. The hysteresis diagram of the unreinforced masonry prism under cyclic-lateral displacement loading is shown in Figure 13b. Due to the overall damage parameters in tension and compression in the CDP material model employed, the stiffness of the URM steadily declines with each cycle of loading.

**Figure 14:**
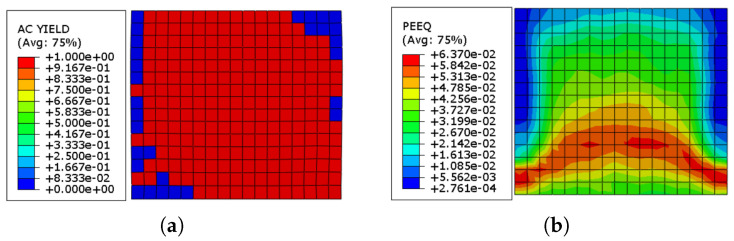
(**a**) AC yield distribution in the URM wall; (**b**) equivalent plastic strain (${\tilde{\varepsilon }}_{c}^{pl}$) distribution in the URM wall.

Figure 14a demonstrates the elements in the URM wall that are yielded at the completion of the cyclic loading. It can be observed that towards the ending of the loading, about 89% of the masonry prism had reached the plastic zone. Figure 14b depicts the equivalent plastic strain (${\tilde{\varepsilon }}_{c}^{pl}$) distribution in the URM at the end of the loading program, with maximum, average, and lowest values $6.370\times 10^{-2}$, $3.466\times 10^{-2}$, and $2.761\times 10^{-4}$, respectively.

**Figure 15:**
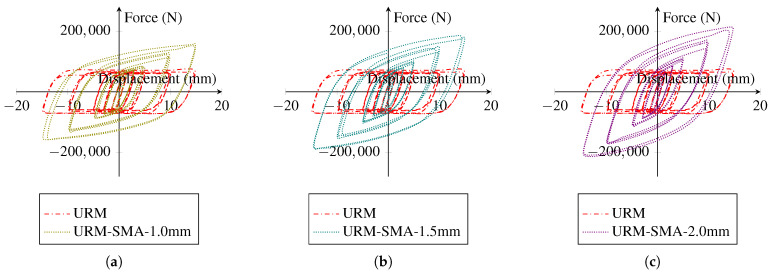
Effect of the variation of the thicknesses value of Ni–Ti pseudoelastic strips on the hysteresis of the masonry wall: (**a**) 1.0 mm; (**b**) 1.5 mm; (**c**) 2.0 mm.

Figure 15 displays the hysteresis response of URM walls reinforced with Ni–Ti strips of different thicknesses in contrast to the URM wall. The application of Ni–Ti strips greatly enhanced the stiffness, energy dissipation (the total area inside the hysteresis curves), and damping capability of the wall, as indicated. In a parametric investigation, it can also be demonstrated that increasing the thickness of the Ni–Ti strip enhances the system’s energy absorption substantially. However, as one of the primary limitations of utilizing Ni–Ti in civil structures is the high manufacturing and implementation costs, an average thickness of 1.5 mm is adopted for further numerical simulations.

**Figure 16:**
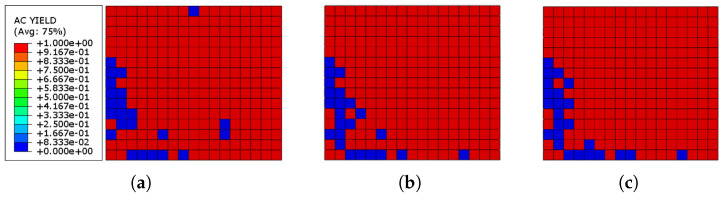
AC yield distribution in the URM reinforced with Ni–Ti strips: (**a**) Ni–Ti thickness = 1.0 mm; (**b**) Ni–Ti thickness = 1.5 mm; (**c**) Ni–Ti thickness = 2.0 mm.

Figure 16 shows the actively yielded elements in the URM wall of the system reinforced with three different thickness values of Ni–Ti strips after the cyclic loading is finished. It can be revealed that after the loading, about 89% of the brick wall in all three models had reached the plastic state, independent of the thickness of the Ni–Ti strips. Furthermore, as the share of yielded components with the systems reinforced with Ni–Ti strips is nearly comparable to the URM wall without any reinforcing element, it can be concluded that the applied Ni–Ti strips do not affect the URM yielding process.

**Figure 17:**
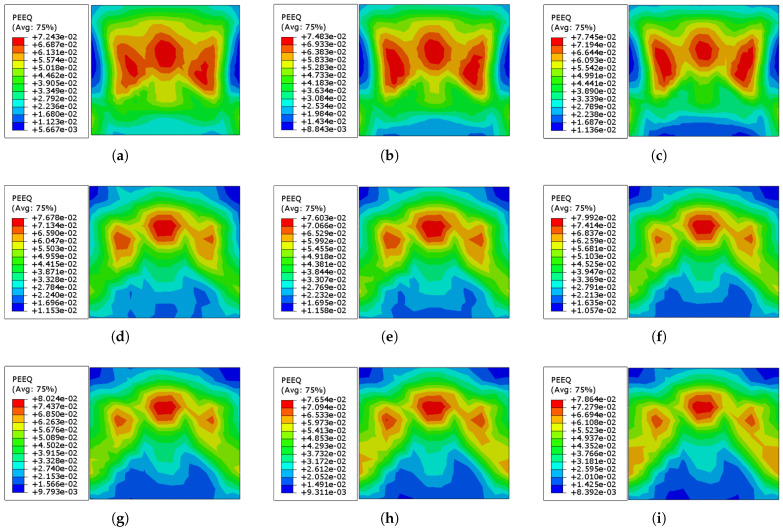
Equivalent plastic strain (${\tilde{\varepsilon }}_{c}^{pl}$) distribution in the URM retrofitted with Ni–Ti strips with different thicknesses at the end of loading program shown in Figure 1: (**a**) Ni–Ti 1.0 mm; (**b**) Ni–Ti 1.5 mm; (**c**) Ni–Ti 2.0 mm; (**d**) Ni–Ti 2.5 mm; (**e**) Ni–Ti 3.0 mm; (**f**) Ni–Ti 3.5 mm; (**g**) Ni–Ti 4.0 mm; (**h**) Ni–Ti 5.0 mm; (**i**) Ni–Ti 6.0 mm.

It is also essential to evaluate if increasing the thickness of the Ni–Ti strips implanted on the wall influences the failure mode and limits the wall’s stiffness. To examine this impact, nine strips with thicknesses ranging from 1 to 6 mm are modeled and mounted on the brick wall, and the results of the nonlinear analysis under cyclic loading are shown in Table 7 and Figure 17.

Table 7 shows that increasing the thickness of the Ni–Ti strips elevated the maximum displacement of the brick wall except for three points where a decrease occurred (2.5, 4.0, and 6.0 mm). Moreover, the maximum values of the Mises stress dropped in the ranges of 1.0–2.0 mm and 4.0–6.0 mm of Ni–Ti thickness, whereas a rise was observed when the thickness was increased from 2.5 to 3.5 mm. Except for three values where a decline occurred (2.5, 3.0, and 5.0 mm), increasing the thickness of the Ni–Ti strips enhanced the maximum plastic strain of the brick wall. Except at the thickness of 4.0 mm, where the values reduced marginally, the dissipated energy rose mainly.

According to the results in Figure 17, increasing the Ni–Ti thickness reduces the average value of the equivalent plastic strain (${\tilde{\varepsilon }}_{c}^{pl}$), improving the overall behavior of the masonry wall under cyclic loading. However, the accumulation of higher values mostly spread from the middle of the wall to the opposite perpendicular sides of the wall.

**Figure 18:**
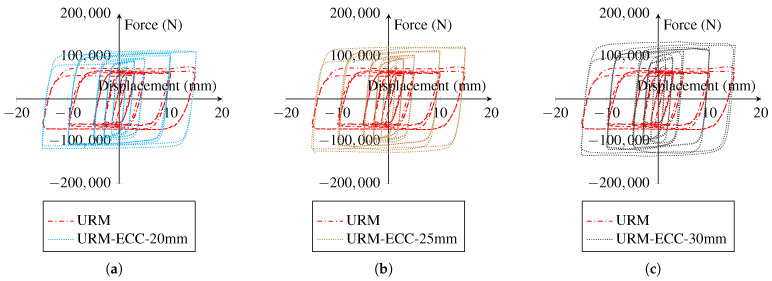
Effect of variation of the thicknesses value of ECC sheet on the hysteresis of the masonry wall: (**a**) 20 mm; (**b**) 25 mm; (**c**) 30 mm.

Figure 18 illustrates the cyclic behavior of URM walls reinforced with ECC sheets of different thicknesses. In comparison to the utilization of Ni–Ti strips, the application of ECC sheets mildly increased the strength and energy dissipation capabilities of the wall. Furthermore, increasing the thickness of the ECC sheet from 20 to 30 mm slightly improved the strength of the wall. As a result, the average thickness of 25 mm was determined for further numerical modeling.

**Figure 19:**
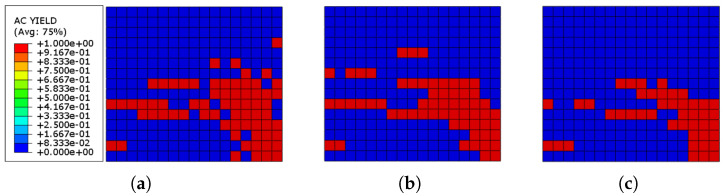
AC yield distribution in the URM reinforced with ECC sheets: (**a**) ECC thickness = 20 mm; (**b**) ECC thickness = 25 mm; (**c**) ECC thickness = 30 mm.

Figure 19 shows the yielded elements in the URM wall of the system reinforced with three different thickness values of ECC sheets connected to the wall through a cohesive contact. It can be revealed that after the loading, about 27%, 24%, and 18% of elements yielded in the models reinforced with ECC sheets with the thickness values of 20, 25, and 30 mm, respectively. Therefore, it can be concluded that ECC sheets efficiently reduce the number of yielded elements in the URM wall and also, the higher the ECC thickness, the less the yielded elements.

**Figure 20:**
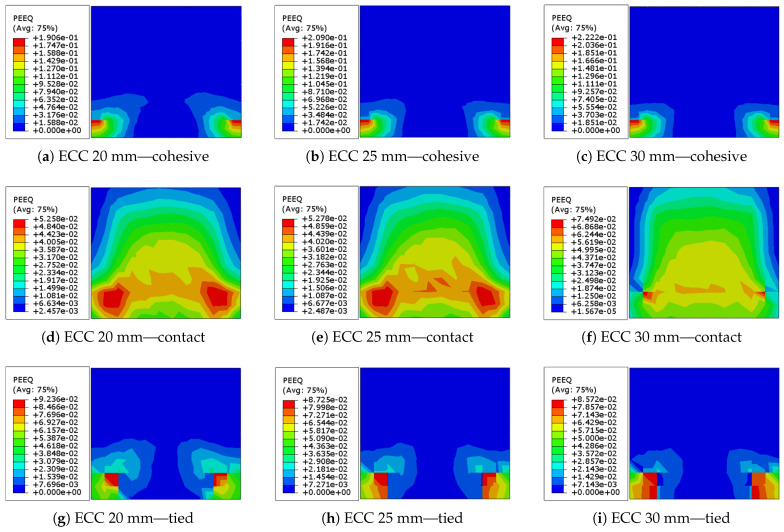
Equivalent plastic strain (${\tilde{\varepsilon }}_{c}^{pl}$) distribution in the URM retrofitted with ECC sheets with different thicknesses and interacting methods at the end of loading program shown in Figure 1: (**a**) ECC thickness = 20 mm with cohesive interaction; (**b**) ECC thickness = 25 mm with cohesive interaction; (**c**) ECC thickness = 30 mm with cohesive interaction; (**d**) ECC thickness = 20 mm with contact interaction; (**e**) ECC thickness = 25 mm with contact interaction; (**f**) ECC thickness = 30 mm with contact interaction; (**g**) ECC thickness = 20 mm with tied constraint; (**h**) ECC thickness = 25 mm with tied constraint; (**i**) ECC thickness = 30 mm with tied constraint.

In the study of Singh and Munjal [35] the optimum thickness of the ECC sheets used for retrofitting the masonry walls is between 20–50 mm. However, to investigate the bond failure of the models by increasing the thickness of the walls from 20 to 30 mm, in addition to the cohesive behavior presented previously, two other interacting approaches connecting masonry prism and ECC sheets are used and the results are compared. A hard contact behavior with a friction coefficient of 0.4 is modeled based on the study by Odacıoğlu and Doğan [54]. In another interacting method, the ECC sheets are connected to the masonry wall by tied constraint. After the analysis of the masonry walls retrofitted with different ECC sheet thickness and interacting methods, the results are compared and presented in Table 8 and Figure 20.

As indicated in Table 8, the dissipated energy in models with cohesive interaction is rather greater than in models with tied constraint and almost twice as high as in models with hard contact. In the models with cohesive behavior and tied constraints, raising the thickness progressively increases the dissipated energy, whereas the model with hard contact shows a modest increase. It is noteworthy that raising the thickness of ECC in the hard contact behavior from 20 to 25 mm marginally increased the maximum displacement of the wall (from 6.703 to 6.765 mm) while increasing it to 30 mm doubled it (13.120 mm). In the model with tied constraint, however, increasing the thickness of ECC from 20 to 25 mm, and subsequently to 30 mm, caused the maximum displacement to decline (from 3.331 to 2.409 mm) and then slightly rise (2.738 mm). The maximum equivalent plastic strain grew by increasing the thickness of the ECC sheets in both models with cohesive and hard contact behavior, whereas it reduced marginally in the model with tied constraint.

Based on the data provided in Figure 20, it can be concluded that increasing the ECC thickness in the models with cohesive behavior and tied constraint has little effect on the overall behavior of the masonry wall under cyclic loading. However, in the model with hard contact behavior, there is a significant variance in the cyclic behavior of brick walls of varying thicknesses. The cyclic behavior of the masonry wall is very similar for walls reinforced with ECC sheets with thicknesses of 20 and 25 mm; however, raising the thickness to 30 mm indicates a substantial variation.

**Figure 21:**
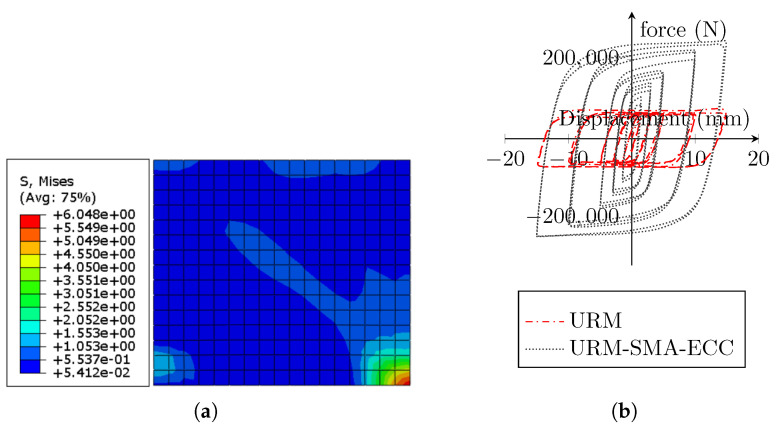
(**a**) Stress distribution in the masonry wall used in the URM-SMA-ECC system; (**b**) hysteresis of the masonry wall.

Figure 21 depicts the hysteresis behavior of a brick wall reinforced with Ni–Ti strips and ECC sheets. In comparison to the prior models of reinforced walls with only Ni–Ti strips and ECC sheets, the composite model with both reinforcing elements yields much larger hysteresis loops, i.e., the area under the curves indicating energy dissipation, compared to the URM wall, is substantially larger in the hybrid model (about 318%).

**Figure 22:**
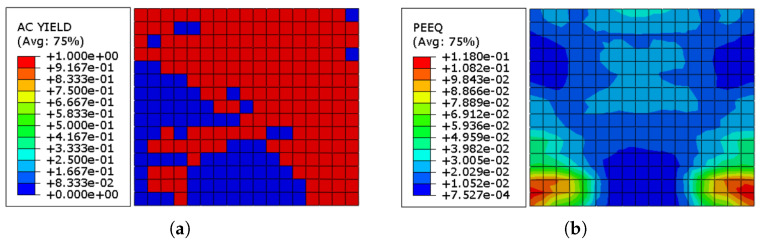
(**a**) AC yield distribution in the URM; (**b**) equivalent plastic strain (${\tilde{\varepsilon }}_{c}^{pl}$) distribution in the URM.

Figure 22a illustrates the yielded components in the URM wall that are produced when the cyclic loading is completed. It can be seen that by the end of the loading, about 68% of the masonry wall is in the plastic state. The equivalent plastic strain (${\tilde{\varepsilon }}_{c}^{pl}$) pattern in the URM at the end of the loading operation is represented in Figure 22b, with maximum, average, and minimum values of $1.180\times 10^{-1}$, $5.277\times 10^{-2}$, and $7.527\times 10^{-4}$, accordingly.

**Figure 23:**
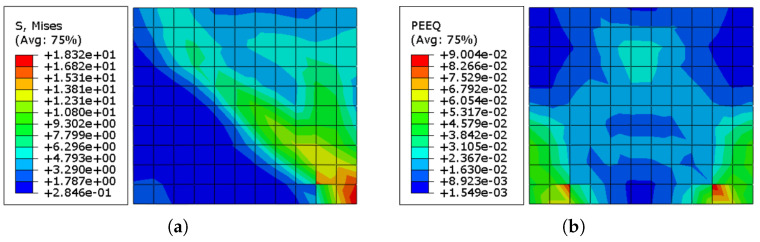
(**a**) Stress distribution in the ECC sheets used in the URM-SMA-ECC system; (**b**) equivalent plastic strain (${\tilde{\varepsilon }}_{c}^{pl}$) distribution in the ECC sheets used in the URM-SMA-ECC system.

Figure 23a presents the residual stress concentration in the ECC sheet. It can be conveyed that the ECC sheet sustained larger stresses than the URM wall, implying that it contributed to the mitigation of wall deterioration. Figure 23b depicts the distribution of equivalent plastic strain (${\tilde{\varepsilon }}_{c}^{pl}$) in the ECC sheets utilized. Although the ECC sheets were subjected to higher stresses, the plastic strain values were much lower due to their greater Young’s modulus compared to the masonry prism.

**Figure 24:**
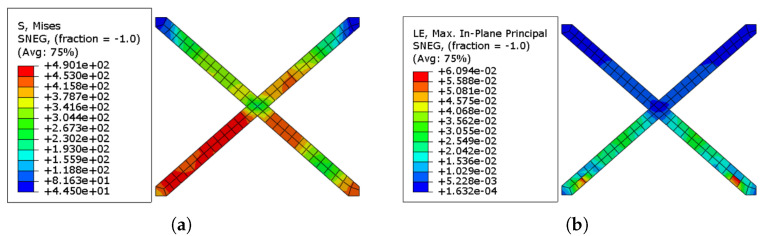
(**a**) Stress distribution in the Ni–Ti Strips used in the URM-SMA-ECC system; (**b**) Strain distribution in the Ni–Ti Strips used in the URM-SMA-ECC system.

The cumulative stress accumulation in the SMA X-shaped strips is shown in Figure 24a. Because of their pseudoelastic capabilities, the Ni–Ti strips were able to withstand far higher stresses than the URM wall and ECC sheets. Figure 24b displays the residual strain pattern in the Ni–Ti strips. The residual strain values in the Ni–Ti strips are significantly lower when compared to other components, such as URM walls and ECC sheets, while withstanding much greater stresses.

**Figure 25:**
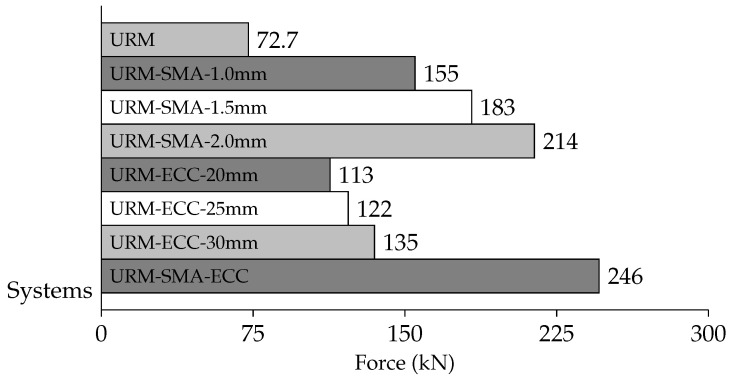
Maximum force at a reference point in each system.

The greatest value of forces at the top of the wall in the reference point was also tested in this investigation, and the findings are shown in Figure 25. When compared to the wall without any reinforcing components, the brick walls strengthened with Ni–Ti strips substantially enhanced the reaction force (RF) values. Furthermore, the walls reinforced with ECC sheets increased the RF value, but at a lesser level than the walls reinforced with Ni–Ti strips. It can be seen that increasing the thickness of both the ECC sheets and the Ni–Ti strips improves the RF values. Among all models, the hybrid model had the highest RF values.

**Figure 26:**
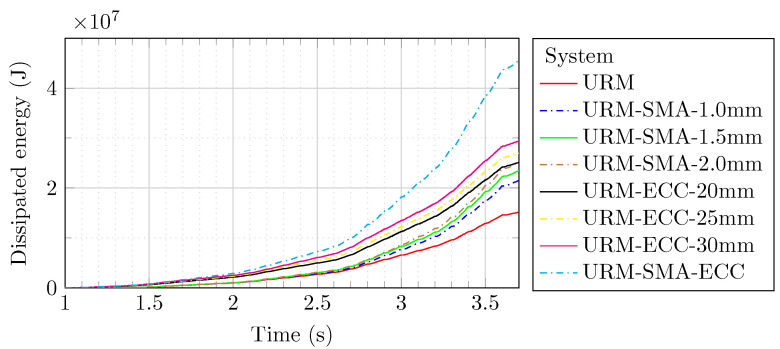
Energy dissipation of different models under cyclic loading.

Under cyclic loading, the quantity of dissipation of energy is proportional to the structure’s transverse stiffness and residual displacement. The results revealed that the hybrid system (URM-SMA-ECC) during the cyclic loading dissipated energy at a level of $4.74\times 10^{7}$ J, which was more than three times that of the URM ($1.52\times 10^{7}$ J) and much greater than the other systems, as shown in Figure 26. It is noticeable that the ECC-based systems absorbed slightly more energy than the system reinforced merely with Ni–Ti strips. Furthermore, when the thickness of ECC sheets and Ni–Ti strips increased, so did the total dissipated energy. As a result, the greater the thickness of the sheets and strips on the brick wall, the greater the dissipation of energy.

## 4. Conclusions

This research examines the numerical simulation of masonry walls reinforced with pseudoelastic Ni–Ti strips and ECC sheets that are exposed to cyclical lateral loading. The material characteristics used in this study have previously been validated through experimental results and published research. In general, the following findings were achieved by installing X-shaped Ni–Ti strips and ECC sheets on brick walls and exposing them to cyclic loads:

- The energy absorption capacity is one of the most essential characteristics of a construction. According to the results, the usage of Ni–Ti strips and ECC sheets significantly boosted this property. When compared to a URM, the total energy dissipated by plasticization in the hybrid system of URM-SMA-ECC increased by 318%.
- It was also discovered that increasing the thickness of ECC sheets has a small effect on hysteresis and very marginally improves it. On the other hand, increasing the thickness of the Ni–Ti strips substantially improved the system’s hysteresis. The hysteresis of the brick walls was marginally improved by increasing the thickness of the ECC sheets and Ni–Ti strips. The hysteresis was raised by 116% by increasing the thickness of the Ni–Ti strips from 1.0 to 2.0 mm. Furthermore, increasing the thickness of the ECC sheets from 20 to 30 mm enhanced the hysteresis by roughly 117%.
- Forces generated during cyclic loading were larger in models reinforced with Ni–Ti strips than in those reinforced with ECC sheets. The produced forces rose from 155 to 214 kN by increasing the thickness of the Ni–Ti strips from 1.0 to 2.0 mm. However, increasing the thickness of ECC sheets from 20 to 30 mm raised the forces from 113 to 135 kN. Furthermore, the hybrid model yielded the highest response force (246 kN).

## Figures and Tables

**Table 1:** Comparison of various properties of Ni–Ti and stainless steel [26].

Property	Unit ${}^{a}$	Ni–Ti ${}^{b}$	Stainless Steel
Density	kg/m${}^{3}$	6450–6500	7850
Corrosion resistance	-	Very good	Fair
Poisson’s ratio	-	0.33	0.265
Elastic modulus	GPa	28–83	190–193
Specific heat capacity	J/kg ${}^{\circ }$C	450–620	420–510
Thermal conductivity	w/m ${}^{\circ }$C	8.6–18	8.9–16.2
Ultimate tensile strength	MPa	895–1900	505
Yield Stress	MPa	70–690	215
Recoverable elongation	%	5–10	0.8
Elongation failure	%	5–50	20

${}^{a}$ The ranges for the presented properties are based on the results of several studies, to provide a wide variety of resources; ${}^{b}$ the range of the given Ni–Ti quantities is influenced by the crystal phase (martensite or austenite) and other parameters (hardened or fully annealed).

**Table 2:** Geometrical information of URM models.

Models	SMA Thickness (mm)	SMA Width (mm)	ECC Thickness (mm)
URM	-	-	-
URM-SMA-1.0 mm	1.0	120	-
URM-SMA-1.5 mm	1.5	120	-
URM-SMA-2.0 mm	2.0	120	-
URM-ECC-20 mm	-	-	20
URM-ECC-25 mm	-	-	25
URM-ECC-30 mm	-	-	30
URM-SMA-ECC	1.5	120	25

**Table 3:** Mechanical properties of Ni–Ti memory alloy presented by Pereiro-Barceló and Bonet [43].

Property	Unit	Value
Young’s modulus (austenite)	MPa	64,647
Young’s modulus (martensite)	MPa	28,125
Poisson’s ratio	-	0.33
$M_{f}$	${}^{\circ }$C	−49.15
$M_{s}$	${}^{\circ }$C	−31.23
$A_{s}$	${}^{\circ }$C	−20.75
$A_{f}$	${}^{\circ }$C	−7.70

**Table 4:** Plastic properties of the homogenized brick wall and the ECC material used in this study.

Material	Dilation Angle (ψ)	Eccentricity (ε)	$\sigma_{b0}/\sigma_{c0}$	K${}_{c}$	Viscosity (μ)
Masonry	30${}^{\circ }$	0.1	1.16	0.667	$1.0\times 10^{-5}$
ECC	37${}^{\circ }$	0.1	1.16	0.667	$1.0\times 10^{-5}$

**Table 5:** Yield stress and corresponding strain values of masonry prism and ECC sheet [34].

Masonry Prism			
Compression stiffening properties		Tension stiffening properties	
Yield stress (MPa)	Inelastic strain	Yield stress (MPa)	Cracking strain
0.95	0.0	0.28	0.0
1.59	0.0002	0.05	0.00022
1.9	0.0003	-	-
2.57	0.0005	-	-
2.83	0.0006	-	-
**ECC Sheet**			
Compression stiffening properties		Tension stiffening properties	
Yield stress (MPa)	Inelastic strain	Yield stress (MPa)	Cracking strain
19.62	0.0	1.45	0.0
28.08	0.0028	1.06	0.0057
36.79	0.0057	0.43	0.0138
49.60	0.0099	-	-
56.89	0.0127	-	-
62.78	0.0170	-	-

**Table 6:** Comparison between the finite element analysis and the experimental results.

	Experimental [35]		Numerical			
Element	Max. Load (N)	Max. Displacement (mm)	Max. Load (N)	Max. Displacement (mm)	Difference in Max. Load (%)	Difference in Max. Displacement (%)
Masonry	15,480	1.02	15,710	1.01	1.47	0.21
ECC	2620	15.87	2850	15.25	8.8	3.91

**Table 7:** Comparison between the mechanical parameters of the walls reinforced with different Ni–Ti thicknesses.

Ni–Ti Thickness	Max. Displacement (mm)	Max. Mises Stress (MPa)	Max. Plastic Strain ($\times 10^{-2}$)	Dissipated Energy (MJ)
1.0 mm	13.23	4.239	7.243	21.5
1.5 mm	14.28	4.062	7.483	23.4
2.0 mm	15.19	3.950	7.745	25.1
2.5 mm	15.17	3.705	7.678	26.9
3.0 mm	15.82	3.791	7.603	29.1
3.5 mm	17.02	3.800	7.992	30.2
4.0 mm	16.97	3.798	8.024	29.9
5.0 mm	21.43	3.786	7.654	32.25
6.0 mm	18.51	3.710	7.864	35.1

**Table 8:** Comparison between the mechanical parameters of the walls reinforced with different ECC thicknesses and interaction.

Interaction	Max. Displacement (mm)	Max. Mises Stress (MPa)	Max. Plastic Strain ($\times 10^{-2}$)	Dissipated Energy (MJ)
ECC-20 mm				
Cohesive	6.264	4.213	19.060	25.1
Contact	6.703	3.362	5.258	13.9
Tied	3.331	3.023	9.236	23.9
ECC-25 mm				
Cohesive	6.395	4.658	20.90	26.9
Contact	6.765	3.296	5.278	13.9
Tied	2.409	3.094	8.725	25.9
ECC-30 mm				
Cohesive	7.543	4.679	22.220	29.3
Contact	13.120	2.293	7.492	14.2
Tied	2.738	3.104	8.572	27.7

## Data Availability

The data presented in this article are available upon request to the corresponding authors.
